# Visfatin and global histone H3K9me levels in colon cancer

**DOI:** 10.1080/07853890.2021.1925737

**Published:** 2021-05-19

**Authors:** Eman A. Al Abdulsalam, Rowyda N. Al Harithy

**Affiliations:** a Department of Biochemistry, College of Science, King Abdulaziz University, Jeddah, Saudi Arabia; b Prince Sultan Military Medical City, Riyadh, Saudi Arabia

**Keywords:** Histones, Western blot, Global histone 3 modifications, Methylation, Epigenetic

## Abstract

**Background:**

Visfatin is considered to be a biomarker in various types of cancers, including colon cancer. Moreover, evidence for epigenetic mechanism must be reported for an association between visfatin level and colon cancer. Therefore, this study was designed to investigate the status of visfatin expression and the global histone three modifications in colon cancerous tissue.

**Methods:**

Colon cancerous tissue and paired adjacent non-cancerous tissue from 30 patients were used to determine the global histone three modifications using Western blot. Quantitative real-time polymerase chain reaction (qRT-PCR) was used to assess visfatin's expression level in tissues.

**Results:**

The visfatin and the global H3K9me expression levels were significantly higher in colon cancerous tissue than in the paired adjacent non-cancerous tissue.

**Conclusion:**

The present study makes a crucial noteworthy contribution to visfatin effect on colon cancer development *via* H3K9me.

## Introduction

Colon cancer is a common and fatal neoplastic disease caused by irregularities in various molecular pathways. According to the World Health Organisation (WHO), in 2018, 1.80 million new colon cancer cases were diagnosed, and 862,000 patients died of the disease globally. To reduce disease incidence and mortality, continuously improving prevention and early detection methods are critical. Therefore, many studies aim to identify colon cancer-critical genes to uncover novel screening and prognostic biomarkers.

Over the last few decades, adipokines have established a reputation for playing a part in carcinogenesis and tumour progression [2–5]. Visfatin is an adipokine that was first discovered as a pre-B-cell colony-enhancing factor (PBEF) that is highly expressed in visceral fat [6]. It has been known as nicotinamide phosphoribosyltransferase (NAMPT), encoded by the *NAMPT* gene, and found to be synthesized and released by a wide variety of tissue types [7,8]. Visfatin has been shown to have endocrine, autocrine, and paracrine functions [9]. Of visfatin’s many functions, it is important to note the part it plays in the production of nicotinamide adenine dinucleotide (NAD), in the activation of the insulin signalling cascade, in increasing glucose uptake, and in inhibiting glucose release [8–10]. Furthermore, visfatin is a regulatory protein in several inflammatory disorders [11–13].

The dysregulation in visfatin concentration has many pleiotropic and pathophysiological effects. Visfatin overexpression was detected in neoplastic tissue and plasma of cancer patients, specifically colon cancer patients [14–18]. Furthermore, visfatin is essential for the NAD + salvage pathway [19,20]. An increase in NAD + derivatives, NADH, and NAD^+^/NADH ratio, were shown to promote colon cancer progression [21].

Recent studies have shown that cancer harbour global epigenetic abnormalities. Therefore, understanding colon cancer progression from an epigenetic perspective will help develop intelligent novel strategies that allow the prevention, diagnosis, and treatment of colon cancer at a molecular level, which reduces mortality and morbidity from the disease itself as from the current treatment methods. Visfatin affects colon cancer development; however, the mechanism is unclear. The present study aimed to explore the association between visfatin expression and the global histone three modifications in colon cancer patients.

## Materials and methods

### Study population

The local ethics committee approved the study at Prince Sultan Military Medical City (PSMMC), Oncology Department Riyadh, Saudi Arabia. The study group included 30 Saudis with colon cancer (19 females age range: Mean ± SEM 58.42 ± 2.79 year and 11 males age range: Mean ± SEM 63.64 ± 3.56 year). All participants provided written informed consent after receiving information about the purpose of the study.

### Sample preparation

Tissue samples were collected from patients during their colectomy procedure. Two samples were collected from each patient, one from the cancerous tumour tissue and adjacent non-cancerous tissue. The distance between cancerous tissue and the adjacent non-cancerous tissue was more than 10 cm. The tissue samples were stored at −80 °C in RNAlater solution (Invitrogen by Thermo Fisher Scientific, USA).

### Total RNA extraction and cDNA

According to manufacturer protocol, total RNA was extracted from tissues using RNeasy Plus Mini Kit (Qiagen, Germany). Assessments of the concentration and the total RNA's quality were carried out using Thermo Scientific NanoDrop 2000c Spectrophotometer (Thermo Scientific, USA). Total RNA (400 ng/sample) was reverse transcribed into cDNA using the Reverse Transcription System kit (Promega, USA).

### Quantitative real-time PCR (qPCR)

To quantify visfatin mRNA expression, a custom TaqMan™ gene expression assay (FAM/MGB probe; Life Technologies, USA) was performed using StepOnePlus™ real-time PCR system. NAMPT (Hs00237184_m1) and GAPDH as housekeeping (Hs99999905_m1) were purchased from Life technologies. A single qPCR reaction was performed in 20 µl volume containing 10 µl of 2x TaqMan gene expression master mix, 1 µl 20x TaqMan gene expression assay, 1 µl of cDNA sample, and 8 µl water free of RNase and DNase. Both targets and internal control were amplified in triplicate in all samples. The reactions were incubated at 50 °C for 2 min, activation step at 95 °C for 10 min, followed by 40 cycles at 92 °C for 15 s, 60 °C for 1 min. The visfatin expression was normalized to GAPDH, and relative expression was determined using the ΔΔCt method.

### Histone protein extraction

Tissue samples were ground using liquid nitrogen and then lysed using 150 µl cold high salt buffer (20 mM Hepes, 0.65 M NaCl, 1 mM EDTA, 0.34 M Sucrose; pH 7.5) that contained fresh Halt Protease and Phosphatase Inhibitor Cocktail at 1X final concentration (Thermo-scientific, USA). Thereafter the lysates were vortexed and stored at −80 °C overnight. The samples were put in a sonicator for 10 cycles; the sonicator (Bioruptor 300, Diagenode from Belgium) was adjusted for 30 s on and 30 s off per cycle. The sample was then centrifuged for 20 min (14,000 rpm) at 4 °C, and the supernatant was kept at −80 °C.

### Western blotting

The concentration of proteins in the samples were measured using the Bradford protein assay. For protein separation, 5 µg was loaded into NuPAGE 4–12% gradient gels (Invitrogen, USA) and ran at 125 volts for 2 h. Upon completing the separation, the proteins were transferred onto a 0.45 µm nitrocellulose membrane (GE Healthcare, Amersham, Germany) using a semi-dry technique at 12 volts for 1 h and 20 min (Trans-Blot SD Cell Bio-Rad, USA). Post-transfer, the membrane was blocked for 30 min using 10% skimmed milk dissolved in tris buffered saline (TBST). After blocking, the membrane was washed with TBST 3 times, and each time was for 10 min. Following washing, the membrane was probed at 4 °C for overnight with primary antibodies (mouse anti β‐actin, 0.3:1000, Abcam #ab8226; rabbit anti H3K27ac, 1:1000, Thermo-fisher scientific #MA5-24671; goat anti H3, 1:1000, Abcam #ab12079; rabbit anti H3K27me2, 1:1000, Thermo-fisher scientific #MA5-11197; rabbit anti H3K9ac, 1:1000, Thermo-fisher scientific #701269; rabbit anti H3K9me2, 1.5:1000, Thermo-fisher scientific #701783; rabbit anti H3K9me, 2:1000, Thermo-fisher scientific #701782; rabbit anti H3K4me2, 1:1000, Thermo-fisher scientific #701764). Blot was washed 3 times for 5 min each with TBST. Then the blot was re-probed with secondary antibody biotin-labelled (Jackson Immune-Research Lab, UK) 1: 10,000 for 1 h at room temperature based on host species. Next, the membrane was washed 3 times for 5 min each with TBST. Another secondary antibody was added to the same blot, streptavidin (Jackson Immune-Research lab, UK) 1: 10,000 for 1 h at room temperature. Finally, the blot was washed 3 times for 15 min each with TBST. According to manufacturer instructions, Super Signal West Pico Chemiluminescent substrate was used for the protein sample image (Thermo Fisher Scientific, USA). Most images were captured manually with 3-5 s exposure and no binning using Amersham Imager 680. All acquired images were analysed using ImageJ software (Rasband, W.S., ImageJ, U. S. National Institutes of Health, Bethesda, Maryland, USA, https://imagej.nih.gov/ij/, 1997–2018). The relative change in the H3 PTM was quantified using the same method detailed by Yang et al.[22].

### Statistical analysis

Statistical analysis was carried out using GraphPad Prism 8.0. (GraphPad Software, La Jolla, CA, USA). Descriptive data were expressed as mean ± standard error of the mean (SEM). To calculate whether visfatin gene expression and histone protein expression levels differed in cancerous colon tissue compared to adjacent non-cancerous tissue, Wilcoxon matched-pairs signed-rank test was used, as the data were not normally distributed. The normality test is based on the D'Agostino-Pearson normality test. A *p*<.05 was considered significant.

## Results

### Visfatin and global histone 3 post-translational modifications expression in Colon tissues

**Figure 1.**
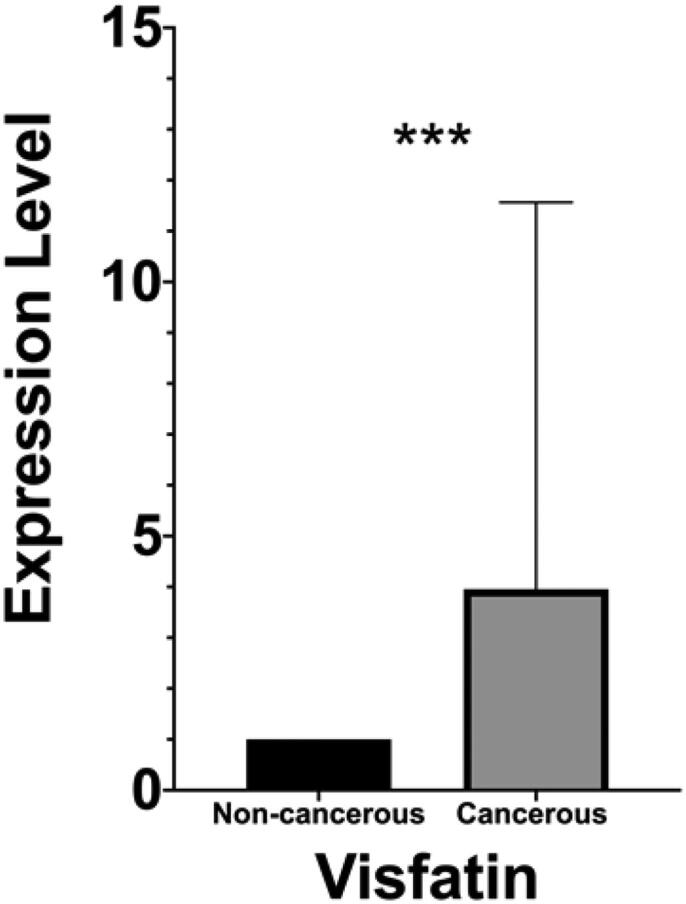
Visfatin expression levels analysis in colon cancerous tissue and paired adjacent non-cancerous tissue. To calculate the difference, non-parametric Wilcoxon matched pairs signed rank test was used. ***, highly significant.

**Figure 2.**
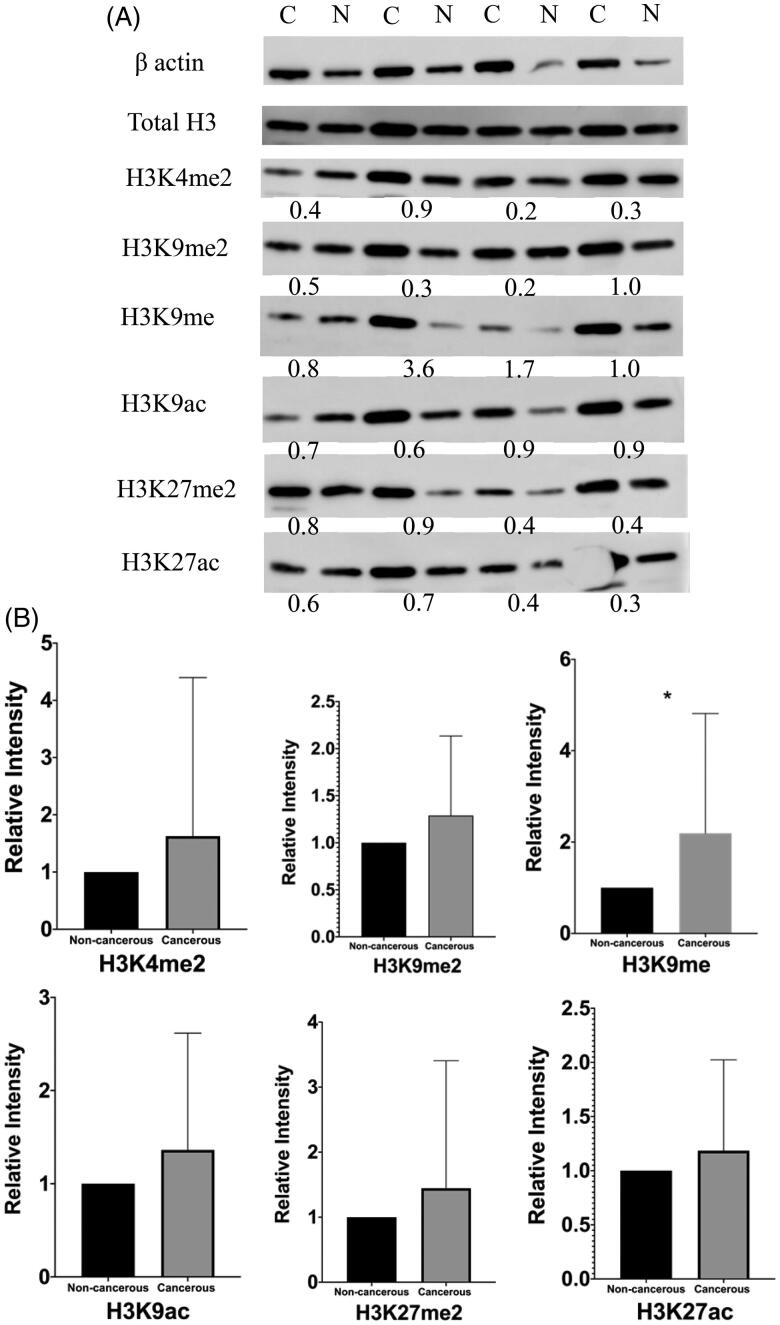
Western blotting of global histone modifications on H3 protein in colon cancerous tissue and paired adjacent non-cancerous tissue. A, six post-translational modifications (H3K4me2, K9me2, K9me, K9ac, K27me2 and K27ac) were measured. β-actin was used as a control. Total H3 was used to validate extraction procedure. The number under each pair of bands in the blots showed relative expression ratios. The ratios <1, =1, and >1 represent decreased, unchanged, or increased expression in cancerous samples, respectively. C, cancerous tissues; N, adjacent non-cancerous tissues. B, columns represent the mean of densitometric values for both cancerous and non-cancerous tissues. H3K9me expression level upregulated in cancerous tissue compared with paired adjacent non-cancerous tissue with *P*-value <0.05.

The gene expression data of a total of 60 samples, including 30 cancerous and 30 paired adjacent non-cancerous, were analyzed using Wilcoxon matched-pairs signed-rank test. The expression level of visfatin was significantly higher (*p* = .0007) in colon cancerous tissue than in non-cancerous tissue (Figure 1). Western blot analysis has revealed the six proteins' expression level: H3K4me2, H3K9me2, H3K9me, H3K9ac, H3K27me2 H3K27ac (Figure 2). The global H3K9me expression level was significantly higher (*p* = .0293) in colon cancerous tissue than in the paired adjacent non-cancerous tissue with *p*-value <.05 (Figure 2). No significant changes were found in the expression levels of H3K4me2, H3K9me2, H3K9ac, H3K27me2, and H3K27ac.

## Discussion

To the best of our knowledge, this is the first study to uncover the contribution of high visfatin level on colon cancer development through an increase of global histone H3K9me. Although colon cancer incidence and mortality are increasing globally, the scientific community continues to uncover the complexity of the disease process. Understanding the mechanisms of its development will allow for early detection and better management of the disease. With the turn of the century, the recognized histone modifications that affect chromatin-based processes continues to expand. In cancer research, efforts have focussed on epigenetic mechanisms that control transcriptional regulation and the corresponding dysregulation in cancer.

Visfatin had become a high-profile topic for its involvement in the progression of many malignant tumours, such as colorectal, ovarian, breast, gastric, and prostate cancers [23,24]. Earlier studies have noted the upregulated level of visfatin in different cancerous tissues, including colon, breast, and malignant astrocytoma cancers [14,25–27]. It has been suggested that visfatin is a biomarker to gastric and colorectal cancers [3,15,27]. Our study mirrors those that preceded us and support that visfatin is a potential biomarker. Furthermore, studies found that the high expression of visfatin in malignant tissue was able to predict poor prognosis in colon cancer and a high level of visfatin in colorectal cancer patient's serum correlated with poor disease staging [3,28,29]. Therefore, visfatin is a useful biomarker and could be clinically relevant in prognosis and staging disease.

Visfatin in mammalian cells was found to be located in both the nucleus and the cytoplasm. Subsequently, the transport of visfatin into the nucleus increases NAD + synthesis involved in hundreds of biochemical reactions. Scientists have uncovered a set of NAD-consuming enzymes that direct how cells behave for processes that include histone modifications [10,30]. NAD + is considered an epigenetic regulator because of its direct involvement with the Sirtuins(histone three lysine deacetylase), poly ADP-ribose polymerase (PARP) in DNA repair mechanism [10], and indirect with C-terminal-binding protein (CtBP). Sirtuins (SIRT1-7) are a deacetylase family of 7 proteins. SIRT1 is well known and has an effect on H3K9 deacetylation [31]. PARP has different effect on H3K4me3, H3K9me2/me3, and H3K27me3 [32]. CtBP is associate with H3K9 demethylase activity [33]. However, inhibition of visfatin (NAMPT) leads to depletion in NAD + and consequently effect sirtuins and PARP. Therefore, visfatin induces epigenetic regulation in histone post-translational modifications (PTMs) [34].

Identifying the NAD-consuming enzymes and their processes helped to understand histone methylation and the complex nature of chromatin regulation. A well-known example of these enzymes is a histone demethylase (LSD1), also known as KDM1A is a lysine-specific demethylase dedicated to removing mono, di-methylation of histone H3 at lysine 4 [35]. Overexpression of LSD1 was found to contribute to human carcinogenesis in various cancers, including colorectal carcinomas [36] and the proliferation and metastasis of colon cancer [37]. This can explain our finding where the expression of H3K9me in cancerous colon tissue is significantly higher than the corresponding adjacent non-cancerous tissue. Our study implies the existence of additional isoforms of histone demethylases that favour mono demethylation at H3K9. While research in the field has provided and revealed many aspects of histone demethylase biology, interesting questions remain to be answered: question one is, whether unidentified LSD1 isoform exist? Another open question is whether the unidentified isoform of histone demethylases regulate colon cancerous tissue differentiation through H3K9me? The central question is whether the unidentified isoform of histone demethylases at H3K9me will be therapeutic targets in the future of “epigenetic medicines.”

Although the current study is a pilot study, it suggests that aberration of the visfatin and the global H3K9me levels is an important epigenetic event associated with colon cancer development. Moreover, offers insight into histone demethylase crucial role that is provoked with visfatin overexpression level. It also addresses the methylation states of histone H3K9me in colon cancer as a biomarker of colon cancer progression and/or prognosis. With every discovery in the epigenetic landscape of tumours, this might suggest a future treatment for colon cancer, knowing that epigenetic changes are possibly reversible. Future perspectives need to investigate visfatin tumour sources and consider the visfatin promoter region's involvement in the histone modification activity. Surely all that will set forward novel concepts for epigenomic studies and help answer the questions outlined in this paper.

## Data Availability

Data are available upon request.
